# An immunoinformatic approach to assessing the immunogenic capacity of alpha-neurotoxins in elapid snake venoms

**DOI:** 10.3389/fphar.2023.1143437

**Published:** 2023-04-10

**Authors:** Yi Wei Chan, Choo Hock Tan, Choon Han Heh, Kae Yi Tan

**Affiliations:** ^1^ Protein and Interactomics Laboratory, Department of Molecular Medicine, Faculty of Medicine, University of Malaya, Kuala Lumpur, Malaysia; ^2^ Venom Research and Toxicology Laboratory, Department of Pharmacology, Faculty of Medicine, University of Malaya, Kuala Lumpur, Malaysia; ^3^ Department of Pharmaceutical Chemistry, Faculty of Pharmacy, University of Malaya, Kuala Lumpur, Malaysia

**Keywords:** three-finger toxins, antivenom efficacy, bioinformatics, immunogenicity, antigenicity, molecular docking

## Abstract

**Introduction:** Most elapid snakes produce venoms that contain alpha-neurotoxins (α-NTXs), which are proteins that cause post-synaptic blockade and paralysis in snakebite envenoming. However, existing elapid antivenoms are known for their low potency in neutralizing the neurotoxic activity of α-NTXs, while the immunological basis has not been elucidated.

**Methods:** In this study, a structure-based major histocompatibility complex II (MHCII) epitope predictor of horse (*Equus caballus*), complemented with DM-editing determinant screening algorithm was adopted to assess the immunogenicity of α-NTXs in the venoms of major Asiatic elapids (*Naja kaouthia, Ophiophagus hannah, Laticauda colubrina, Hydrophis schistosus, Hydrophis curtus*).

**Results:** The scoring metric M_2_R, representing the relative immunogenic performance of respective α-NTXs, showed all α-NTXs have an overall low M_2_R of <0.3, and most of the predicted binders feature non-optimal P1 anchor residues. The M_2_R scores correlate strongly (R^2^ = 0.82) with the potency scores (p-score) generated based on the relative abundances of α-NTXs and the neutralization potency of commercial antivenoms.

**Discussion:** The immunoinformatic analysis indicates that the inferior antigenicity of α-NTXs is not only due to their small molecular size but also the subpar immunogenicity affected by their amino acid composition. Structural modification with conjugation and synthetic epitope as immunogen may potentially enhance the immunogenicity for improved antivenom potency against α-NTXs of elapid snakes.

## 1 Introduction

Alpha-neurotoxins (α-NTXs) belong to the three-finger toxins (3FTx) family and are commonly found in the venoms of most Asiatic elapid snakes, e.g., cobras (*Naja* spp.), sea snakes (*Hydrophis* spp.), King Cobra (*Ophiphagus hannah*) and kraits (*Bungarus* spp.) (Tan et al., 2015a; Tan et al., 2015b; Tan et al., 2015c; Leong et al., 2015; Oh et al., 2021). Depending on the amino acid composition, the α-NTXs are further classified into short neurotoxins (SαNTX, 6–7 kDa) and long neurotoxins (LαNTX, 7–9 kDa) (Barber et al., 2013). As antagonists of post-synaptic nicotinic acetylcholine receptors (nAChR) of neuromuscular junctions, these non-enzymatic neurotoxins account for the neurotoxicity in most elapid envenomation, resulting in a fast onset of flaccid systemic paralysis, with death ensuing rapidly in the absence of proper treatment (Barber et al., 2013; Ranawaka et al., 2013).

Antivenoms, which contain animal immunoglobulins or derivatives raised against snake venoms, remain the definitive treatment for snakebite envenoming (World Health Organization, 2016). However, earlier studies consistently showed elapid antivenoms have low efficacy, with neutralization potency (P, expressed in the amount of venom neutralized completely per milliliter of antivenom) well below 1 mg/ml (Leong et al., 2012; Leong et al., 2015). In contrast, viperid antivenoms appear to have higher efficacy, with Potency (P) values up to 10 mg/ml (Leong et al., 2014). The lower efficacy of elapid antivenoms would inevitably lead to the need for a higher dose of antivenom treatment, resulting in higher medical expenses, curtail of antivenom stockpile, and at the same time exposing patients to a greater risk of hypersensitive reactions that can be fatal (World Health Organization, 2016; Patikorn et al., 2022). The elapid antivenom efficacy is particularly low in neutralizing venom lethality driven by α-NTXs (Ratanabanangkoon et al., 2016; Wong et al., 2016; Tan et al., 2017), suggesting inadequate immunogenic capacity of the α-NTX for the production of neurotoxin-specific antibodies. In this regard, factors such as the size, dose and foreignness of a molecule are known to affect its immunogenicity as an antigen during immunization (Clementi et al., 1991; Irais et al., 2020). The small size of α-NTX indicates a lower number of epitopes in eliciting an immune response, and the immunogenicity may be further compromised by other larger snake venom toxins present in an immunogen mixture. To overcome the current predicament, the concept of “diverse toxin repertoire” has been proposed and trialled whereby the pool of α-NTXs of various elapid species was used to enrich the venom immunogen mixture containing minimum high molecular weight (MW) components for hyperimmunization in horses (Ratanabanangkoon, 2021). The resultant antisera showed an exceptionally broad para-specificity of neutralization against diverse elapid venoms, while the strategy did not translate into a major improvement of neutralizing potency per individual snake species (Ratanabanangkoon et al., 2016; Ratanabanangkoon et al., 2020). Furthermore, even antivenoms raised against elapid venoms that contain abundant α-NTX (>40% of total venom proteins) showed generally low neutralizing potency, questioning if the “dose” of α-NTX is indeed the key determinant of eliciting good immunization response (Ratanabanangkoon et al., 2016; Palasuberniam et al., 2021; Tan et al., 2021). Hypothetically, there are other factors at play that intrinsically modulate the makeup of epitope(s) in the α-NTX, and this results in variable immunorecognition by the B-cell receptor (BCR) and/or T-cell receptor (TCR) for antibody production.

Conventionally, antivenom production exploits the adaptive immune system of host animals such as horses, in which venom proteins are used as specific antigens to strategically mount a humoral immune response. This entails the activation and maturation of naïve B-cells into antibody-producing plasma B-cells, under the regulation of helper T cells (also called CD4^+^ cells). To produce specific venom-targeting antibodies, the venom antigens must first be recognized, bound and internalized by professional antigen-presenting cells (APC) which include the naïve B-cells (*via* the BCR), and other phagocytes, e.g., macrophages and dendritic cells. The endocytosed venom proteins are degraded in the endo-lysosomal compartments, yielding peptide fragments as antigens that will be loaded onto major histocompatibility complex II (MHCII) and displayed on the external cell membrane. The MCHII-loaded peptide antigens displayed on phagocytes, especially dendritic cells, will be recognized by naïve T helper cells (T_h_ cells) *via* the TCR, activating them into effector and memory T_h_ cells. The effector T_h_ cells recognize the particular antigen peptide displayed by the MCHII complex of B-cells, fully activating them to produce toxin-specific antibodies (immunoglobulin G) with longer half-life and higher affinity, while the same T_h_ cells also release cytokines that mediate proliferation of the activated B-cells (Hoogeboom and Tolar, 2016).

All in all, initiating the venom/toxin-specific humoral immunity requires proper loading of the toxin peptides onto MHCII membrane-bound proteins for subsequent antigen presentation to the T_h_ cells. The MHCII protein is composed of two subunits (α and β) chains, whose α_1_ and β_1_ domains of the extra-cellular segments form the antigen-binding groove with multiple pockets destined to bind antigenic peptides (of the exogenous toxins) in the endosomal compartment. From the endoplasmic reticulum where MHCII proteins are assembled, throughout their intracellular transport to the Golgi apparatus and finally to endosomes, the MHCII binding groove is blocked from endogenous peptide binding by the invariant (Ii) chain. In the endosomes, the drastic change in pH partially degrades the MHCII-bound Ii chain while leaving behind a short peptide named CLIP (class II MHC associated Ii peptide) that continues to shield the MHCII binding site. Here, the equine version of human leukocyte antigen-DM (HLA-DM) molecule plays a critical role for peptide exchange, where it accelerates the removal of CLIP and other peptides that lack appropriate anchor residues, thereby “editing” the repertoire of internalized peptides to make way for proper MHCII-peptide loading and subsequent antigen presentation on APCs (Morris et al., 1994; Denzin and Cresswell, 1995; Fung-Leung et al., 1996; Zhou et al., 2009; Schulze et al., 2013). The susceptibility to DM-mediated peptide editing is modulated by the dynamic conformation of MHCII bound with peptide. Previous studies highlighted the importance of the hydrogen bond network formed between peptide backbone and conserved residues in MHCII, especially those at the P1 and P2 pockets which are key sites of peptide binding (Stratikos et al., 2004; Anders et al., 2011; Yin et al., 2014). Accordingly, only strong-binding peptides with appropriate anchor residues for the pockets will succeed in the DM-mediated MHCII peptide exchange, demonstrating immunodominance of epitopes over others that favors their recognition by T_h_ cells in mounting a good humoral immune response. In this context, the understanding of the DM-editing susceptibility of various peptides display of α-NTXs to proper MHCII-peptide loading is important to elucidate the immunogenicity of these venom proteins, which has deep implication in antivenom production. Therefore, the present study aimed to assess immunogenic capacity of α-NTXs by using the modified structure-based predictor in complement with the screening algorithm of DM-editing determinants. In addition, a scoring metric was configured to determine the relative immunogenicity of α-NTXs. It is hoped that this study will provide a deeper understanding about the poor efficacy of elapid antivenoms.

## 2 Materials and methods

### 2.1 Prediction of B-cell antigenic determinant

The full amino acid sequence of respective α-NTXs was acquired from UniProt and submitted to the input panel for B-cell epitope prediction with Kolaskar and Tongaonkar antigenicity scale (http://tools.iedb.org/bcell/) (Kolaskar and Tongaonkar, 1990). Selected α-NTXs for this study are: (i) LαNTXs, which include (a) α-cobratoxin (Nk_LαNTX-α-Cbtx, UniProt ID: P01391, *Naja kaouthia*); (b) long neurotoxin OH55 (Oh_LαNTX-OH55, UniProt ID: Q53B58, *O. hannah*); and (c) long neurotoxin 1 (Hc_LαNTX-1, UniProt ID: Q8UW29, *Hydrophis curtus*), and (ii) SαNTXs: (a) cobrotoxin-c (Nk_SαNTX-Cbtx-c, UniProt ID: P59276, *N. kaouthia*); (b) short neurotoxin 1 (Hs_SαNTX-1, UniProt ID: P68415, *Hydrophis schistosus*); and (c) short neurotoxin 2 (Lc_SαNTX-2, UniProt ID: P10457, *Laticauda colubrina*). Zinc metalloproteinase-disintegrin-like atragin (Na_SVMP, Uniprot ID: D3TTC2, *Naja atra*) was selected as a standard of high MW toxin.

### 2.2 Homology modeling of Eqca-DR MHCII models

The homology structures of horse (*Equus caballus*) major histocompatibility complex II (Eqca-DR) were modeled after human HLA-DR1 crystal structure [PDB_ID: 1HXY] obtained from Protein Data Bank (https://www.rcsb.org). With HLA-DR1 selected as a template, molecular modeling was carried out for the two allelic variants of Eqca-DR (Eqca-DRA*00103:DRB2*00201 and Eqca-DRA*00201:DRB2*00201; sequence obtained from EMBL’s European Bioinformatics Institute (https://www.ebi.ac.uk) and modeled *via* webserver Swiss-Model (https://www.swissmodel.expasy.org). The 3D protein models were generated after transferring conserved atom coordinates as identified by the target-template alignment. To acquire a full-atom protein model, the construction of non-conserved amino acid side chains was performed with OpenStructure computational structural biology framework and ProMod3 modeling engine. Both models with the highest Global Model Quality Estimation (GMQE) and optimal QMEAN scores were submitted to Structural Analysis and Verification Server of UCLA (https://saves.mbi.ucla.edu/), and passed the Verify3D (at least 80% of the amino acids have scored ≥0.2 in the 3D/1D profile) and PROCHECK (Ramachandran plot: 92.4% residues in most favored regions, 6.9% residues in additional allowed regions, 0.3% residues in generously allowed regions and 0.3% residues in disallowed regions) assessments (Laskowski et al., 1993; Eisenberg et al., 1997).

### 2.3 Preparation of 15-mer α-NTXs peptide fragments

15-mer peptide sequences were generated automatically with an in-house Bash script that extracts 15 continuous amino acid residues from respective α-NTXs. Following that, the 3-dimensional structures of 15-mer were constructed with Pymol (Schrödinger and DeLano, 2020). Open Babel software was used to minimize the energy of the resulting 15-mer peptide structures with a conjugated algorithm for 100 cycles (O'Boyle et al., 2011).

### 2.4 Molecular docking simulation

The series of docking analyses were performed *via* the guru interface of the HADDOCK 2.2 webserver (https://alcazar.science.uu.nl/services/HADDOCK2.2/). The HADDOCK protocol encompasses three docking stages: (i) rigid body minimization (it0), (ii) semi-flexible annealing (it1), and (iii) explicit solvent refinement (water) (van Zundert et al., 2016). The decoys generated were clustered with Fraction of Common Contacts algorithm (FCC: 0.6) (Rodrigues et al., 2012; Takemura and Kitao, 2019). Cluster with the lowest HADDOCK scores of the top 4 models was ranked on top. By default, the HADDOCK scoring function settings are for protein-protein complexes that entail weighted sum of following terms:

$$HADDOCKscore-it0=0.01\;E_{vdw}+1.0\;E_{elec}+1.0\;E_{desol}+0.01\;E_{air}-0.01\;BSA$$

$$HADDOCKscore-it1=1.0\;E_{vdw}+1.0\;E_{elec}+1.0\;E_{desol}+0.1\;E_{air}-0.01\;BSA$$

$$HADDOCKscore-water=1.0\;E_{vdw}+0.2\;E_{elec}+1.0\;E_{desol}+0.1\;E_{air}$$

where E_vdw_ is the van der Waals energy, E_elec_ is the electrostatic energy, E_desol_ is the desolvation energy, E_air_ restraints (i.e., distance) violation energies, BSA is the buried surface area. More comprehensive HADDOCK protocol and different docking parameter settings were reported in de Vries et al. (2010).

#### 2.4.1 Docking parameters optimization and validation of Eqca-DR MHCII models

To optimize input parameters of HADDOCK, hemagglutinin (HA) [PDB_ID: 1HXY] crystal structure was selected as the standard ligand (used in all quantitative analysis); endogenous peptide A2 (A2) [PDB_ID: 1AQD] crystal structure was selected as reference ligand (used to establish arbitrary binding threshold for binders classification). Both ligands were docked to both allelic variants of Eqca-DR due to their experimental proven MHCII binding (Stern et al., 1994; Murthy and Stern, 1997). The intermolecular interactions of the resulting Eqca-DR:HA and Eqca-DR:A2 complexes were assessed for hydrogen bonding interactions, *via* BIOVIA Discovery Studio Visualizer, between the main-chain peptide and conserved HLA-DR1 residues as reported earlier (Stern et al., 1994; Murthy and Stern, 1997). The sampling size across rigid body minimization (It0), semi-flexible annealing (It1) and explicit solvent refinement (water) stages was optimized at 5000:300:300; random exclusion of ambiguous interaction restraints (AIRs) was disabled. The conserved residues of membrane-distal alpha-helices and whole peptides were selected as input for active residues; passive residues of receptor and ligand were defined automatically by HADDOCK. The computed linear combination of various energies and buried surface area of the Eqca-DR:HA and Eqca-DR:A2 complexes in the form of arbitrary HADDOCK scores were recorded.

#### 2.4.2 Selection of 15-mer α-NTXs peptide fragments by docking with Eqca-DR

The energy-minimized 15-mer peptide fragments derived from α-NTXs were respectively docked onto Eqca-DRA*00103:DRB2*00201 and Eqca-DRA*00201:DRB2*00201 as per Section 2.4.1. The resulting Fraction of Common Contacts algorithm (FCC)-clustered decoys were screened and selected for susceptibility to DM-catalyzed peptide editing based on the defined algorithms in the present study:

1) The backbone atoms of P-1, P1, P2 anchor residues of the examined peptides must be hydrogen-bonded to Serα53-Asnβ82 of MHCII molecules.
2) The examined peptide N-terminus must extrude from the P1 pocket.
3) Bidentate hydrogen bonds must be formed between Asn82β and P2 anchor residue of the examined peptides.
4) The combined count of hydrogen bonds and salt bridges formed between membrane distal alpha-helices of MHCII molecules and the examined peptides must be ≥14. This is to enrich the pool of epitopes featuring binding stability and appropriate poses.

The lowest HADDOCK score of respective MHCII-peptide complexes was computed and inverted bars were plotted. The HADDOCK score of binders filtered *via* the algorithm outlined above was highlighted.

#### 2.4.3 Determination of immunodominant regions of α-NTXs

Considering the immune response is elicited based on the binding strength and successful intermolecular interactions of a complex, the immunodominant regions of α-NTXs were estimated by HADDOCK score along with the scoring of amino acid residues of 15-mer α-NTXs peptides from immunogenic binders. The M_2_R scoring metric, which represents the degree of computed immune response elicited by respective 15-mer peptides in the context of MHCII, was quantified as described in the formulation:

$$M_{2}R=\sum_{n=0}^{I^{\prime }}S_{n+1}$$ (1)

$$S=\frac{\sum_{n=0}^{i}{\mathrm{hr}}_{n+1}}{\sum_{n=0}^{I}{H\prime R\prime }_{n+1}}$$ (2)

Where “*S*” is the score of individual residue constituting α-NTXs, “*I’”* is the total number of amino acid residues constituting α-NTXs, “r” is the frequency of individual amino acid residue that constitutes the filtered 15-mer peptides, “*R’*” represents the maximum frequency attainable for each residue, “*i*” is the total number of MHCII-bound 15-mer peptides, “*I*” is the total number of 15-mer peptides constructed from respective α-NTXs, “*h*” is the HADDOCK score ratio of respective algorithm-filtered decoys and standard ligand (HA), and “*H*’” is the maximum attainable HADDOCK score ratio.

The cumulative score of each residue was quantified, accumulated and plotted to quantify M_2_R which represents the immunogenic spectrum of individual α-NTX.

## 3 Results and discussion

### 3.1 Molecular size and antigenicity of α-NTXs

**FIGURE 1:**
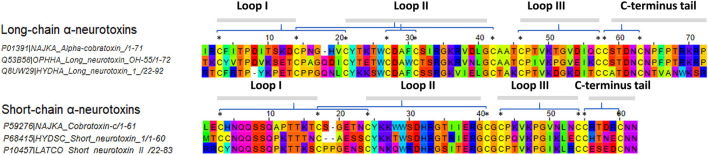
Multiple sequence alignment of selected elapids long-chain and short-chain alpha-neurotoxins. Sequences were acquired from the UniProt database. Abbreviations: *NAJKA*, *Naja kaouthia*; *OPHHA*, *Ophiophagus hannah*; *LATCO*, *Laticauda colubrina*; *HYDSC*, *Hydrophis schistosus*; *HYDHA*, *Hydrophis curtus*. Asterisk symbols indicate the location of disulfide bonds.

**FIGURE 2:**
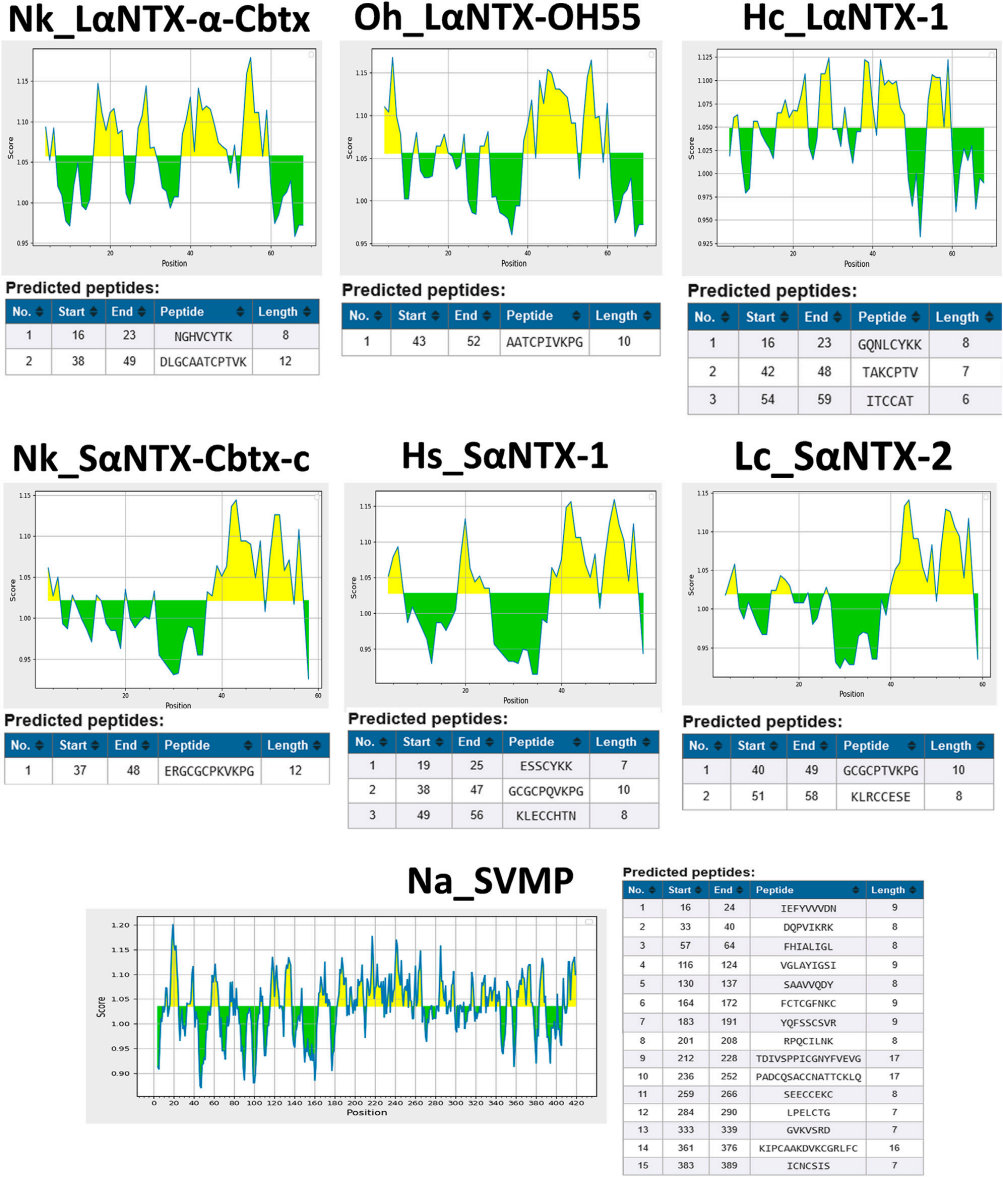
Prediction of antigenic determinants (B-cell epitope) of α-NTXs with Kolaskar and Tongaonkar antigenicity scale (Kolaskar and Tongaonkar, 1990). Abbreviations: Nk_LαNTX-α-Cbtx, α-cobratoxin (UniProt ID: P01391, *N. kaouthia*); Oh_LαNTX-OH55, long neurotoxin OH55 (UniProt ID: Q53B58, *O. hannah*); Hc_LαNTX-1, long neurotoxin 1 (UniProt ID: Q8UW29, *H. curtus*); Nk_SαNTX-Cbtx-c, cobrotoxin-c (UniProt: P59276, *N. kaouthia*); Hs_SαNTX-1, short neurotoxin 1 (UniProt ID: P68415, *H. schistosus*); Lc_SαNTX-2, short neurotoxin 2 (UniProt ID: P10457, *L. colubrina*); Na_SVMP, snake venom zinc metalloproteinase (Uniprot ID: D3TTC2, *Naja atra*) (high MW toxin). Yellow areas represent predicted B-cell epitopes with antigenicity scores above the dynamic threshold (∼1.0).

In the present study, six different lethal elapid α-NTXs (Figure 1) belonging to short-chain and long-chain groups, and a snake venom metalloproteinase [SVMP, a higher molecular weight (MW) toxin] were analyzed for their antigenicity using the B-cell epitope prediction tool (Kolaskar and Tongaonkar, 1990). As shown in Figure 2, the sheer number of predicted antigenic sites of SVMP (MW∼47 kDa, 15 antigenic sites identified) far exceeded that of the six α-NTXs (MW∼7–8 kDa, 1–3 antigenic sites identified), consistent with previous findings of low MW toxins possessing less antigenic peptides (Tan et al., 2019b; Tan and Tan, 2021). The higher number of antigenic peptides in high MW proteins may provide greater exposures to BCRs, thus eliciting a higher antibody titer specific to these antigens, in comparison to the low MW proteins where α-NTXs are classified (MW∼7–8 kDa). Moreover, the presence of high MW proteins in an immunogen mixture containing proteins with varying MW may dampen the antigenicity of low MW α-NTXs (<10 kDa) (Clementi et al., 1991; Irais et al., 2020) in eliciting immune responses amid the antigenic competition for BCRs (Hunt et al., 1995), consequently, the resulting neutralizing antibodies may be skewed toward high MW toxins. This is well exemplified by previous immunoprofiling studies applying microarrays (Engmark et al., 2016) and enzyme-linked immunosorbent assay (Lingam et al., 2020), as well as *in vivo* neutralization studies that showed viperid antivenoms are significantly more potent than elapid antivenoms (Calvete et al., 2016; Ratanabanangkoon et al., 2016; Bailon Calderon et al., 2020; Faisal et al., 2021). This is likely due to most viperid venoms are predominated with high MW toxins (MW > 25 kDa) (e.g., SVMP and snake venom serine protease, SVSP) (Offor et al., 2022), which are capable of eliciting stronger immune response for the production of neutralizing antibodies. However, given that venoms constituting neurotoxins of more than ∼40% failed to raise antivenoms with major improvement in neutralization efficacy (Tan et al., 2015b; Tan et al., 2016a; Tan et al., 2019a; Palasuberniam et al., 2021), it is therefore suggested that the low neutralizing efficacy of elapid antivenom may be also attributed to other factors, such as amino acid composition, that intrinsically modulate the epitope configuration of α-NTXs to be recognized by T-cell receptor (TCR) to elicit a strong humoral response.

### 3.2 Screening of HLA-DM exchange determinant

The present study generated homolog models of Eqca-DR due to the absence of equine MHCII alleles binding data in the currently available databases. The immunogenicity of α-NTXs derived from the venoms of selected Asiatic elapids was examined by docking 15-mer peptides of α-NTXs generated to Eqca-DR, to assess for MHCII epitopes. The use of a structure-based approach in the present study is mainly due to the limitations of the existing range of bioinformatics tools (i.e., NetMHC-3.4, PSSM, SMM, TEPITOPE) in the provision of an accurate estimate on MHCII epitopes. Despite the availability of data-driven avenue, most of the data-driven machine-learning mediated predictors of MHC epitopes were trained with MHC-peptidome composed of synthetic sequences (Sidney et al., 2013) that sidestep proteasome cleavage and DM-editing selection. Although this method could narrow down the potential candidate of MHC epitopes, but it may not provide accurate prediction (Robbins et al., 2013; Yadav et al., 2014). Furthermore, although recent development entails the adoption of naturally processed and presented mass spectrometry (MS) derived MHC peptides to hone the accuracy of the existing predictors, the current MS-derived peptidome datasets still do not correlate well with predicted binding affinity (Zhao and Sher, 2018). Factors such as the indirect linear relationship between the kinetic stability of MHCII-peptide complexes and alignment issues due to peptide length varieties may have bugged the algorithm from performing at full capacity in predicting the MHCII binder (Belmares et al., 2002; Nielsen et al., 2010; ten Broeke et al., 2013). Thus, these limitations raise the need for an alternative avenue for immunogenicity study.

**FIGURE 3:**
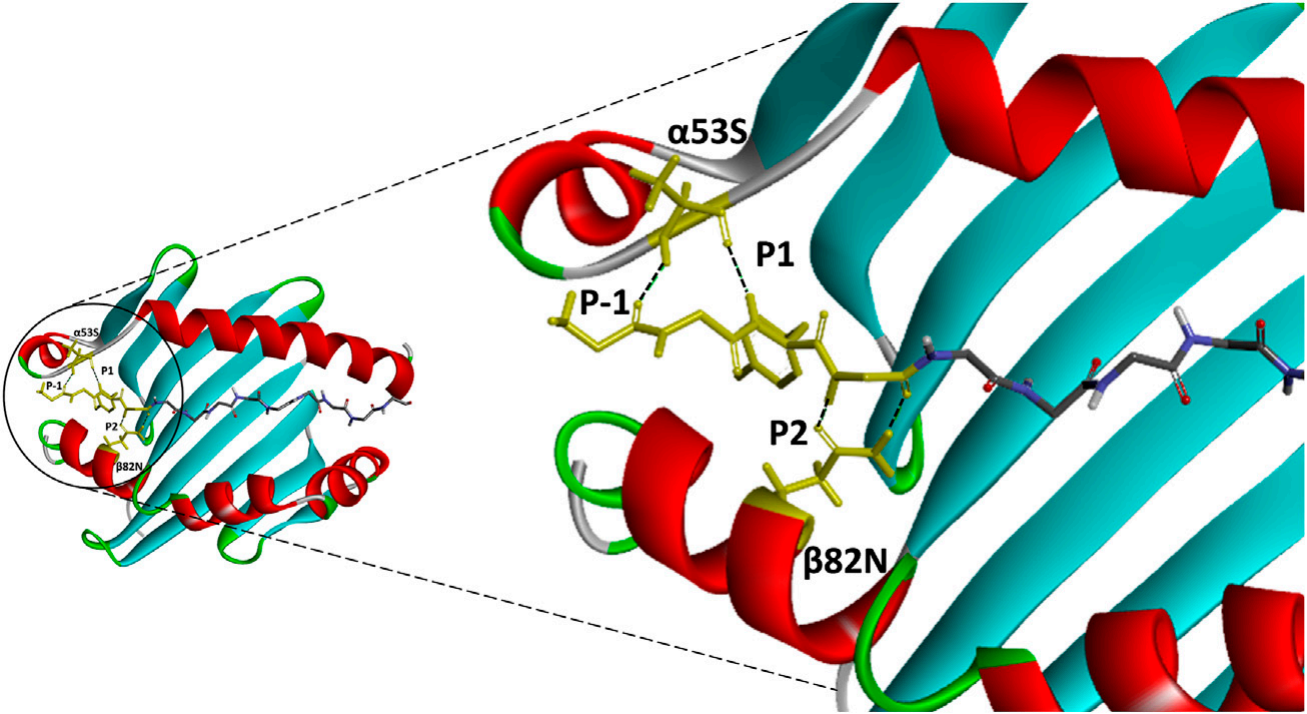
An atomic resolution of HLA-DR1:HA complex derived from PDB crystal structure (PDB:1HXY) with annotated critical intermolecular interactions between peptide ligand and the side chains of MHCII. Peptide backbone that formed conserved hydrogen bonds with MHCII residues (α53S and β82N) were denoted as P-1, P1 and P2 anchor residues and highlighted in yellow.

**FIGURE 4:**
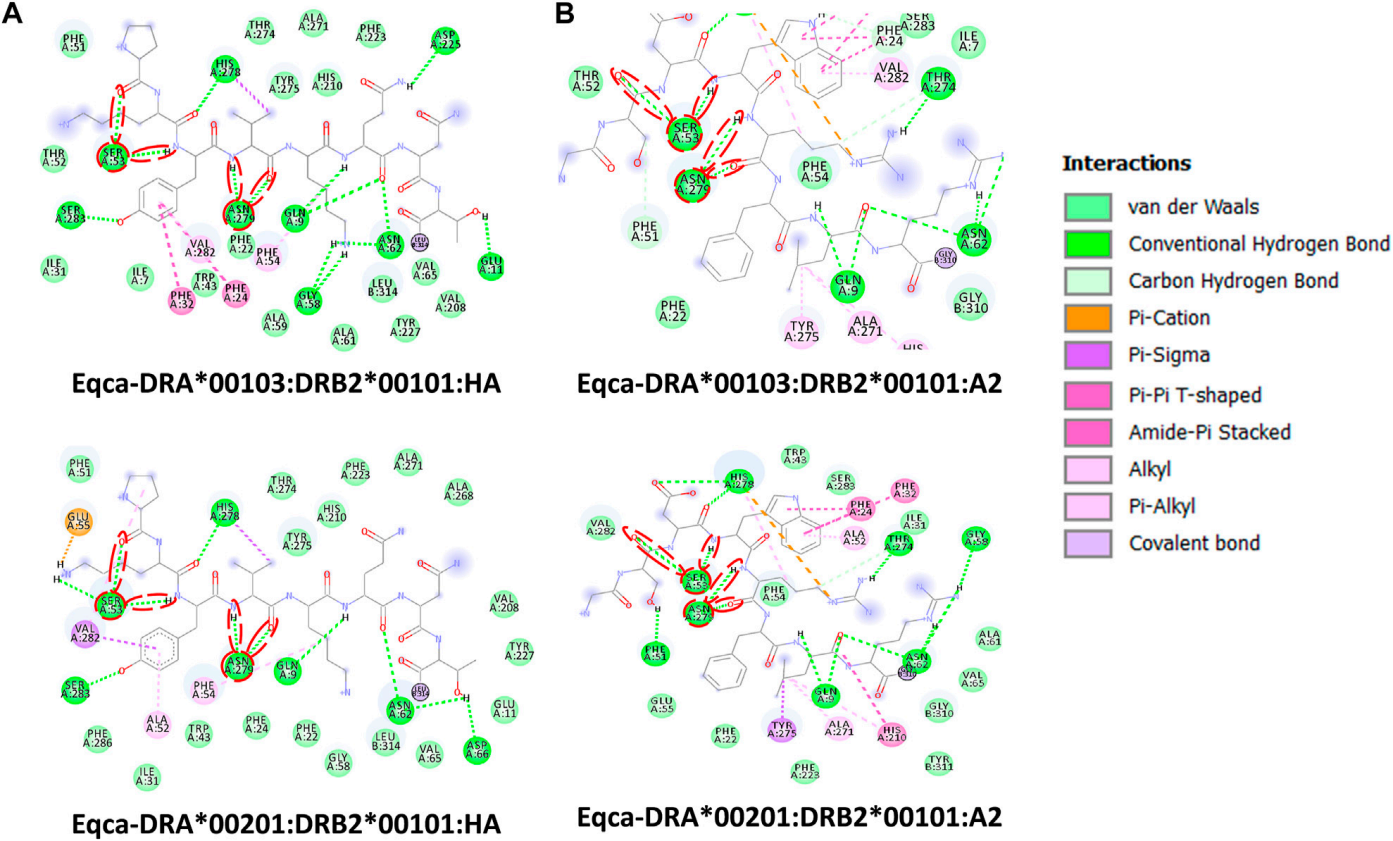
Molecular interactive illustration showing the interaction of redocked standard ligands with homology models of the two allelic Eqca-DR. **(A)** hemagglutinin (HA) and **(B)** endogenous peptide A2 (A2). 2D chemical structure represents the ligand surrounded by interactive MHCII binding groove residues. P-1, P1 and P2 anchor residues of HA and A2 and their hydrogen bonding interaction with residues of the binding grooves were circled in red. Two allelic Eqca-DR models examined were Eqca-DRA*00103:DRB2*00101 and Eqca-DRA*00201:DRB2*00101.

Contrary to the data-driven predictor, the structure-based approach does not rely on binding data. Adding to that, it provides visualization of atomic-resolution intermolecular interaction between the MHCII-peptide complex. Peptides are bound to MHCII *via* hydrogen bonding network between conserved MHCII side chains and peptide backbone and through interactions between MHCII pockets and peptide side-chain anchors. The complementing screening algorithm was customized based on the identification of multiple critical hydrogen bonds between conserved MHCII side-chains and peptide backbone (Figure 3) as a gauge to the susceptibility of DM-catalyzed peptide editing (McFarland et al., 2001; Stratikos et al., 2004; Zhou et al., 2009; Ferrante and Gorski, 2010; Anders et al., 2011; Painter et al., 2011). Following the same principle, MHC-loading enhancers (MLE) target P1 pocket as canonical MLE site to enhance T_h_ cells response, by maintaining the peptide-receptive conformation of MHCII which in turn expedited antigen-loading and ligand exchange on the cell surface (Gupta et al., 2008; Dickhaut et al., 2009). Validation of the MHCII homologs and optimization of docking simulation parameters were conducted by redocking standard (HA) and reference ligands (A2) with two allelic variants of Eqca-DR models (Figure 4). Two homologs examined feature critical intermolecular hydrogen bonding formed between MHCII residues with HA and A2 peptides (with side chains occupying major pockets, hence designated as P-1, P1 and P2 anchor residues), which verified the compatibility of the generated Eqca-DR models and the optimized docking simulation parameters for this study.

Herein, structure-based MHCII epitopes predictor augmented with DM-editing determinant algorithm, mimicry of humoral response, was adopted to assess the immunogenicity of α-NTXs derived from venoms of selected terrestrial, submarine and marine Asiatic elapids (Figure 1). The use of α-NTXs selected from cross-genus elapids was to validate the flexibility of the structural approach in determining the immunogenicity of α-NTXs, as well as for comparison to the previously reported data from neutralization studies (Tan et al., 2016b; Tan et al., 2018).

### 3.3 Algorithm clustering of MHCII binders

**FIGURE 5:**
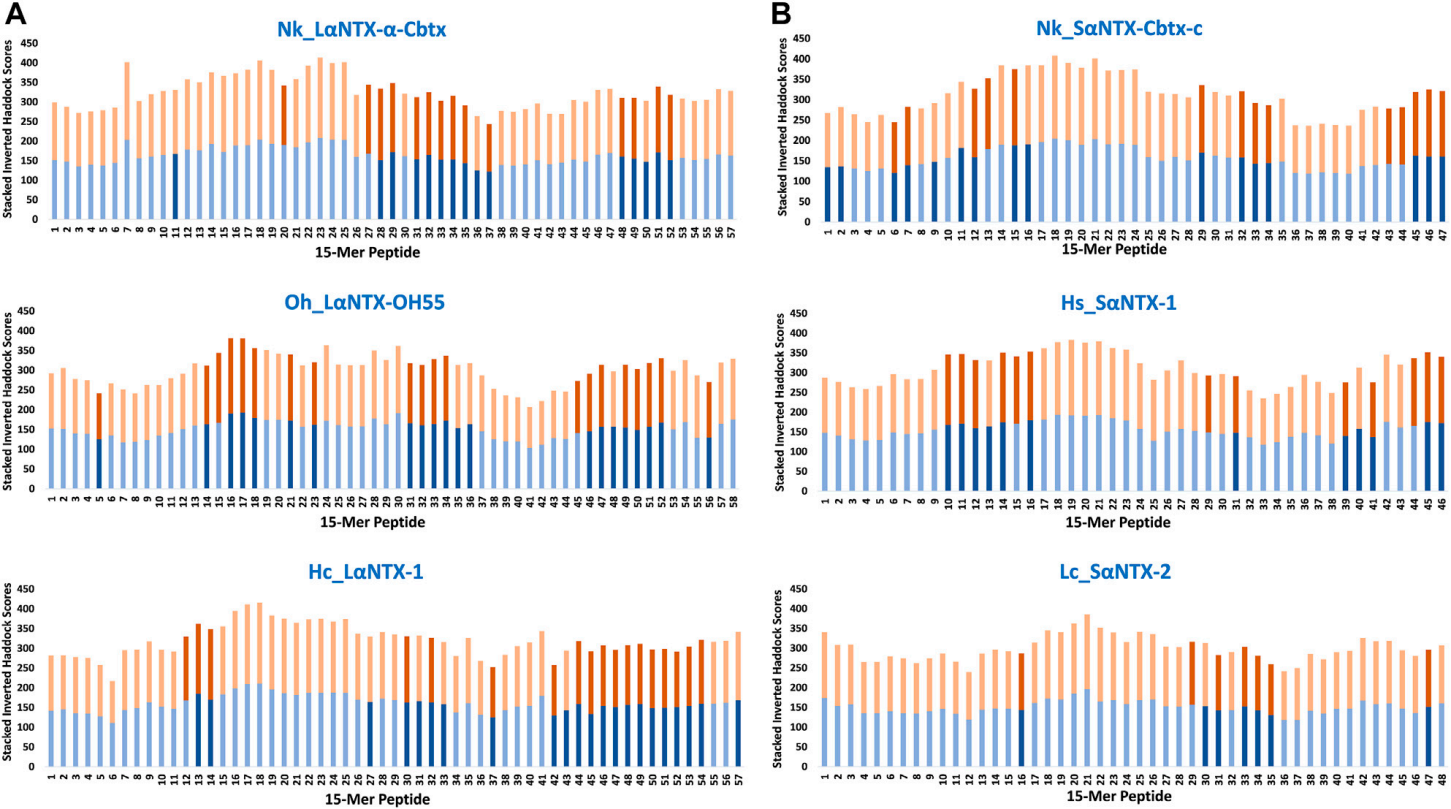
Accumulative inverted HADDOCK scores of 15-mer peptides binding decoys (Eqca-DR:15-mer) derived from docking of Eqca-DRA MHCII models to 15-mer peptides generated from α-neurotoxins. **(A)** Long-chain alpha-neurotoxins (LαNTX), and **(B)** Short-chain alpha-neurotoxins (SαNTX). Blue bars indicate Eqca-DRA*00103:DRB2*00201; and orange bars indicate Eqca-DRA*00201:DRB2*00201. Bars with dark blue/dark brown indicate the decoys (Eqca:15-mer) that fulfilled the defined algorithm in the present study. Abbreviations: Nk_LαNTX-α-Cbtx, α-cobratoxin (UniProt ID: P01391, *N. kaouthia*); Oh_LαNTX-OH55, long neurotoxin OH55 (UniProt ID: Q53B58, *O. hannah*); Hc_LαNTX-1, long neurotoxin 1 (UniProt ID: Q8UW29, *H. curtus*); Nk_SαNTX-Cbtx-c, cobrotoxin-c (UniProt: P59276, *N. kaouthia*); Hs_SαNTX-1, short neurotoxin 1 (UniProt ID: P68415, *H. schistosus*); and Lc_SαNTX-2, short neurotoxin 2 (UniProt ID: P10457, *L. colubrina*).

The arbitrary binding affinity threshold is commonly used as a gauge for the prediction of MHCII epitope ligands (Sette et al., 1994; Southwood et al., 1998). Table 1 showed the HADDOCK score of decoys of two allelic Eqca-DR models (Eqca-DRA*00103:DRB2*00201 and Eqca-DRA*00201:DRB2*00201) redocked with HA and A2 ligands that provide inference for a strong binder (Stern et al., 1994; Murthy and Stern, 1997). Applying the same approach, the inverted stacked HADDOCK scores of decoys of 15-mer peptides with respective allelic variants of Eqca-DR were shown in Figure 5. As delineated by Table 2, the 15-mer peptides were further benchmarked arbitrarily as strong (≤−230), moderate (−230 to −180) and weak binders (≥−180) based upon the HADDOCK score of HA and A2 ligand bindings (Table 1).

**TABLE 1:** HADDOCK scores of redocking of two allelic variants of Eqca-DR MHCII models with standard and reference ligand.

MHCII alleles Ligand	Eqca-DRA*00103:DRB2*00201	Eqca-DRA*00201:DRB2*00201
Hemagglutinin (HA)	−197.4 ± 6.7	−200.2 ± 3.9
Endogenous peptide A2 (A2)	−235.1 ± 0.6	−236.4 ± 2.5

**TABLE 2:** Clustering of display and non-display decoys (Eqca-DR:15-mer) based on HADDOCK scores.

Peptide 15-mers	Eqca-DRA*00103:DRB2*00201			Eqca-DRA*00201:DRB2*00201		
	Binder classification^a^	Predicted MHCII epitope^b^	Predicted MHCII epitope (%)^c^	Binder classification^a^	Predicted MHCII epitope^b^	Predicted MHCII epitope (%)^c^
Long-chain alpha-neurotoxins (LαNTX)						
Nk_LαNTX-α-Cbtx	(S0:M12:W45)	W15	26.32	(S0:M12:W45)	M1W13	24.56
Oh_LαNTX-OH55	(S0:M3:W55)	M2W19	36.21	(S0:M3:W55)	M2W18	34.48
Hc_LαNTX-1	(S0:M12:W45)	W22	38.60	(S0:M9:W48)	W18	31.58
Short-chain alpha-neurotoxins (SαNTX)						
Nk_SαNTX-Cbtx-c	(S0:M12:W35)	M3W13	34.04	(S0:M11:W36)	M1W13	29.79
Hs_SαNTX-1	(S0:M6:W40)	W12	26.10	(S0:M5:W41)	W13	28.26
Lc_SαNTX-2	(S0:M2:W46)	W6	12.50	(S0:M2:W46)	W6	12.50

^a^ 15-mer peptides were classified into “strong binder” (S, HADDOCK score ≤−230), “moderate binder” (M, HADDOCK score −230 to −180), and “weak binder” (W, HADDOCK score ≥−180) based on the arbitrary binding thresholds. Thresholds are defined based on the HADDOCK scores of standard and reference ligands (hemagglutinin and endogenous peptide A2). The numerical values indicate the number of binders.

^b^ 15-mer peptides that fulfilled the defined algorithms outlined in the present study (Section 2.4.2). The numerical values indicate the number of binders.

^c^ Relative percentage of 15-mer peptides with predicted MHCII epitopes over the total number of 15-mer peptides generated per examined α-NTX.

The present study showed none of these 15-mer peptides generated from all α-NTXs were considered as “strong binder.” Out of the 57–58 15-mer peptides generated from the three LαNTXs, there were only 3–12 that were considered as “moderate binders” while the remaining were “weak binders.” Similarly, the SαNTXs that are shorter in sequence length also showed no “strong binders” among the 46–48 15-mer peptides. At this point, the pool of low affinity 15-mer binding MHCII decoys already foreshadows weak immunogenicity, because it is suggested that the peptide that binds with a long half-life has the propensity to dominate the T_h_ cell response (Pos et al., 2013). Although previous studies suggested that MHCII binders with higher affinity may not be translated to MHCII binding *in vivo* (Zhao and Sher, 2018), this may be a result of excluding the importance of peptide-editing role of DM molecules in the prediction or study performed (Belmares et al., 2002).

With the DM-editing determinant screening algorithm used in this study, the filtered 15-mer peptides were selected as predicted MHCII epitopes (Table 2). In comparison, LαNTXs generally feature a higher percentage of predicted MHCII binders (24%–38%) than SαNTXs (12%–34%) (Table 2). Of the LαNTXs derived 15-mer peptides, only 14–22 were predicted as MHCII epitopes, with the lowest binder proportion shown in Nk_LαNTX-α-Cbtx (24%–26%), followed by Oh_LαNTX-OH55 (∼34–36%), and Hc_LαNTX-1 (31%–38%). For SαNTXs, the MHCII binder proportion was ranging from 12% to 34%, with the lowest shown in Lc_SαNTX-2 (∼12%), followed by Hs_SαNTX-1 (26%–28%), and Nk_SαNTX-Cbtx-c (30%–34%). Unsurprisingly, most predicted MHCII binders were on the “weak binder” spectrum, with only a few classified as “moderate binders.”

Binding affinity relative to algorithm-identified binders is suggested as a vital indicator that represents the degree of posing fit which indirectly reflects the importance of amino acid composition, and consequently dictates the dynamic conformation of MHCII-peptide that is least susceptible to DM-editing mechanism (Pos et al., 2013; Yin et al., 2014). Also, it relays information on the possible disposition of P9 leucine-binder in sidestepping the hydrogen bonding and pocket interaction at P1 (Yin et al., 2014), which may be a perk of strong MHCII binder (i.e., endogenous peptide A2). Ergo, it is suggested that the low amount of MHCII epitopes and their relatively weak affinity may compromise the chances of α-NTXs to be processed for MHCII display by APCs that non-specifically uptake and process antigens for the activation of T_h_ cell in the context of humoral immunity (Hilhorst et al., 2014).

#### 3.3.1 Binding specificity of major anchor sites of MHCII

Compositional analysis of α-NTXs was performed by profiling the amino acid residues of predicted MHCII binders derived from respective α-NTXs occupying P-1, P1 and P2 binding pockets of Eqca-MHCII. The known major anchor sites are P1, P4, P6 and P9 as evidenced by their substantial buried side chain solvent-exposed surface area (Murthy and Stern, 1997). However, we specifically look into the P1 pocket as the residue preference in this position plays a major role in dictating DM-catalyzed peptide editing susceptibility relative to other major pockets (Painter et al., 2011).

**FIGURE 6:**
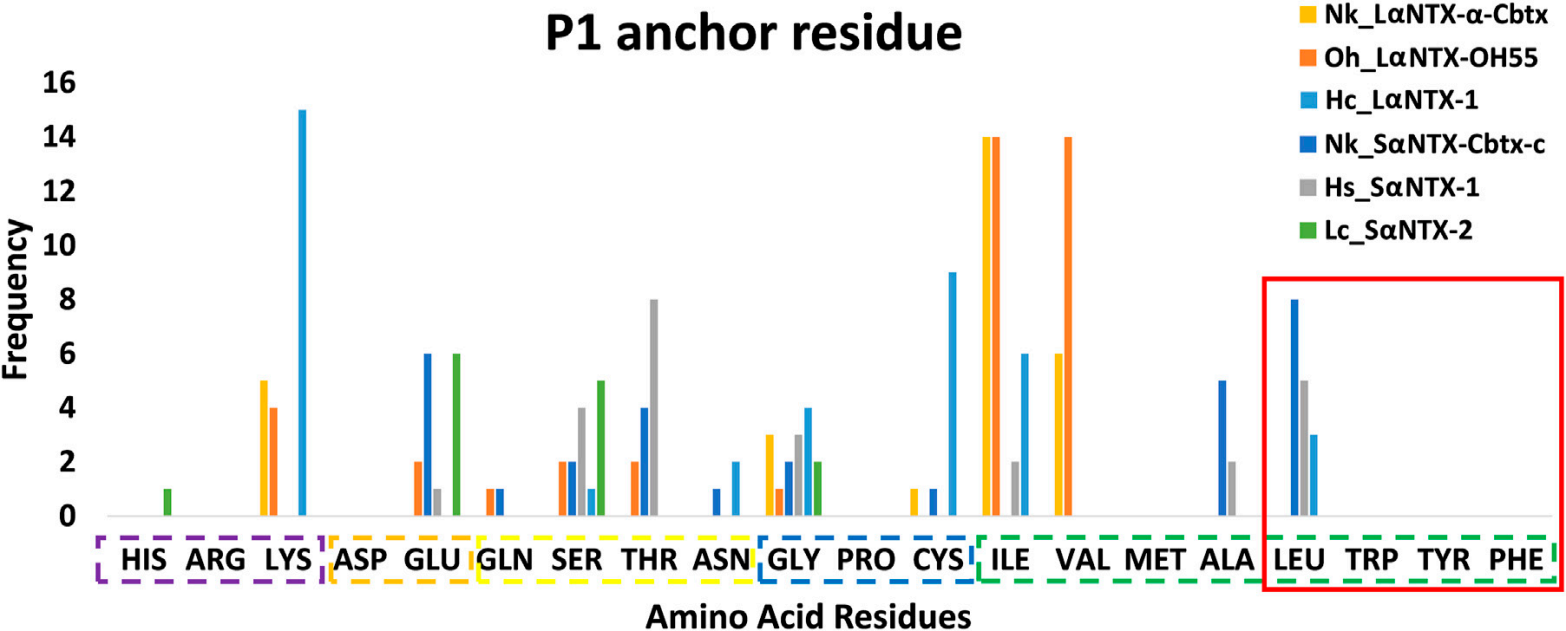
Anchor residue preferences of P1 pocket. Violet box: Positive side chain; Orange box: Negative side chain; Yellow box: Polar side chain; Blue box: Special cases; Green box: Non-polar side chain; Red square: Optimal P1 anchor residues. Abbreviations: Nk_LαNTX-α-Cbtx, α-cobratoxin (UniProt ID: P01391, *N. kaouthia*); Oh_LαNTX-OH55, long neurotoxin OH55 (UniProt ID: Q53B58, *O. hannah*); Hc_LαNTX-1, long neurotoxin 1 (UniProt ID: Q8UW29, *H. curtus*); Nk_SαNTX-Cbtx-c, cobrotoxin-c (UniProt: P59276, *N. kaouthia*); Hs_SαNTX-1, short neurotoxin 1 (UniProt ID: P68415, *H. schistosus*); and Lc_SαNTX-2, short neurotoxin 2 (UniProt ID: P10457, *L. colubrina*).

An earlier study showed tryptophan, tyrosine, phenylalanine and leucine that feature hydrophobic side chains are optimal P1 anchor residues (Yin et al., 2014). As depicted in Figure 6, only 16 out of 177 (<10%) identified predicted MHCII binders feature optimal P1 anchor amino acid residues mentioned above. These include Nk_SαNTX-Cbtx-c (8 binders), Hs_SαNTX-1 (5 binders), and Hc_LαNTX-1 (3 binders), and worth noting that all 16 binders consistently possess leucine as anchor residue. The lack of optimal P1 anchor residues may have culminated in binders with weak affinity to Eqca-DR which coincides with the predominant of weak MHCII binders as displayed in Table 2. Thus, this finding further validates that the amino acid composition of α-NTXs could be one of the major limiting factors in eliciting a strong humoral response, during the immunization process of antivenom production.

### 3.4 Immunodominance of α-neurotoxins

**FIGURE 7:**
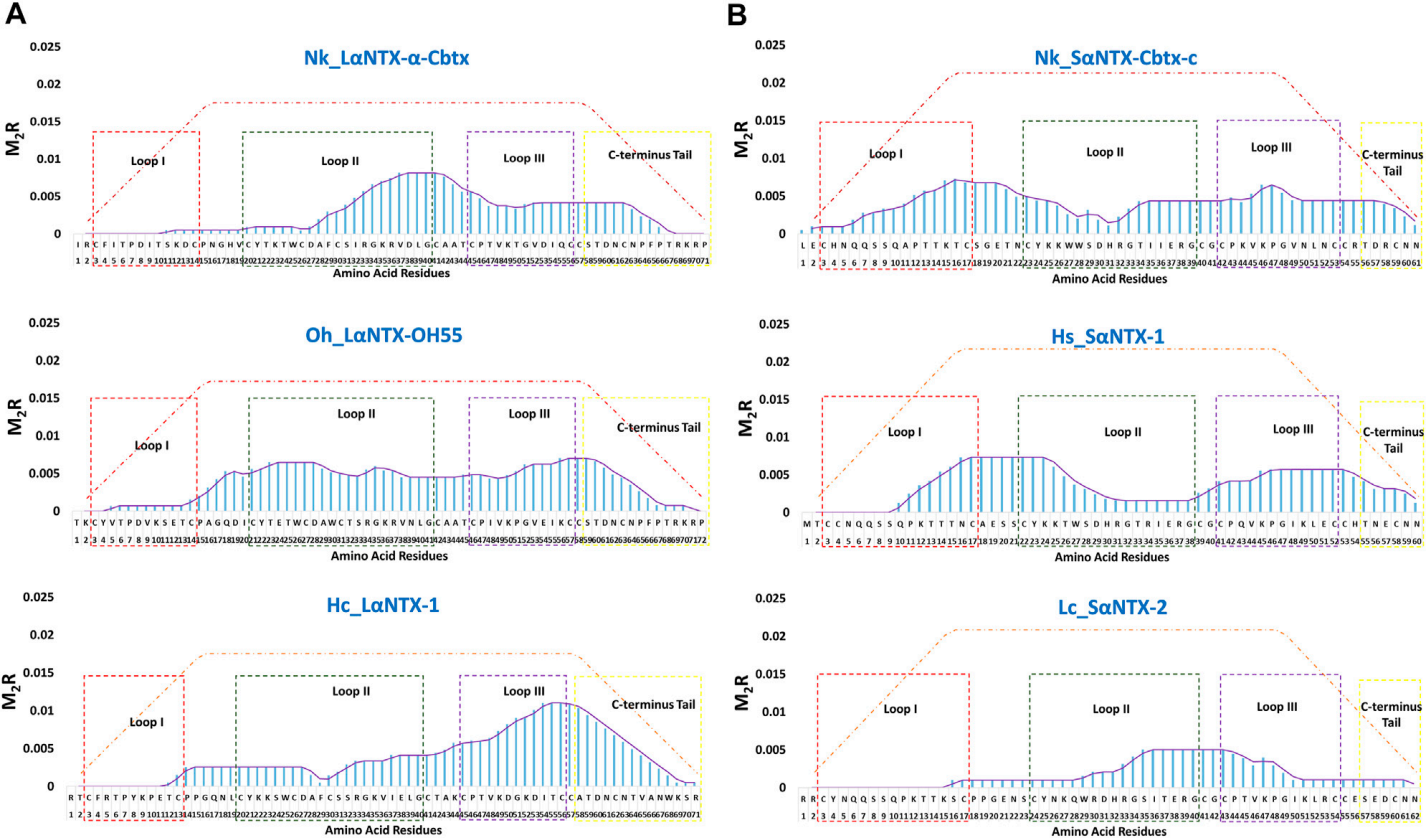
The immunodominant coverage across the amino acid sequences of α-neurotoxins. **(A)** Long-chain alpha-neurotoxins (LαNTX), and **(B)** Short-chain alpha-neurotoxins (SαNTX). Dashed squares in the figure represent the different loops and C-terminus tail of α-NTXs. The brown-dash dotted line indicates the maximum score of respective amino acid residues. Abbreviations: Nk_LαNTX-α-Cbtx, α-cobratoxin (UniProt ID: P01391, *N. kaouthia*); Oh_LαNTX-OH55, long neurotoxin OH55 (UniProt ID: Q53B58, *O. hannah*); Hc_LαNTX-1, long neurotoxin 1 (UniProt ID: Q8UW29, *H. curtus*); Nk_SαNTX-Cbtx-c, cobrotoxin-c (UniProt: P59276, *N. kaouthia*); Hs_SαNTX-1, short neurotoxin 1 (UniProt ID: P68415, *H. schistosus*); and Lc_SαNTX-2, short neurotoxin 2 (UniProt ID: P10457, *L. colubrina*). The details analyses were provided in Supplementary File S1.

For ease of interpretation, scoring metric that reflects both binding affinity and proportion of predicted MHCII binders are vital for multilayered analysis. Based on the predicted MHCII epitopes, each amino acid composing α-NTXs was scored and cumulatively quantified as M_2_R to enable elucidation of immunodominance, in the context of T_h_ cell activation, across the entire sequence of α-NTXs (Figure 7; Supplementary File S1).

In general, the immunodominant amino acid sequences were noted to be skewed differently for all three LαNTXs from different species, where Nk_LαNTX-α-Cbtx and Oh_LαNTX-OH55 were shown to be more immunogenic at loop II, and to a lesser extent for Hc_LαNTX-1 that is more loop III dominant. All three α-NTXs feature minute responsiveness at loop I as indicated by their extremely low M_2_R score. On the contrary, Nk_SαNTX-Cbtx-c, Hs_SαNTX-1 and Lc_SαNTX-2 are responsive across the three loops, except that Lc_SαNTX-2 featured a negligible response for loop I. Overall, all α-NTXs were predicted to be relatively responsive on loop II, which corroborates with a previous study that employed a data-driven MHCII epitope predictor utilizing human and mouse MHCII alleles binding data in predicting the immunogenicity of snake 3FTXs (Pruksaphon et al., 2022).

**FIGURE 8:**
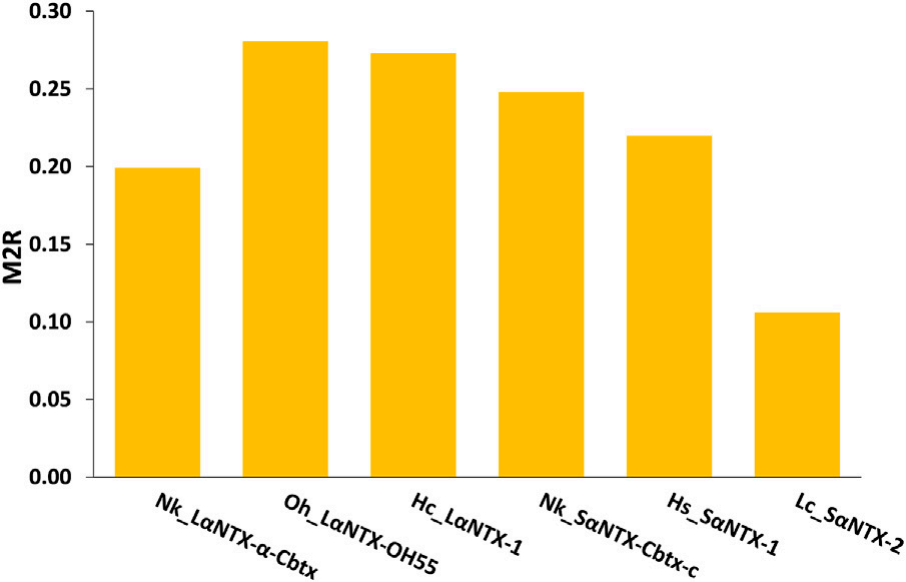
M_2_R scoring of α-neurotoxins. Abbreviations: Nk_LαNTX-α-Cbtx, α-cobratoxin (UniProt ID: P01391, *N. kaouthia*); Oh_LαNTX-OH55, long neurotoxin OH55 (UniProt ID: Q53B58, *O. hannah*); Hc_LαNTX-1, long neurotoxin 1 (UniProt ID: Q8UW29, *H. curtus*); Nk_SαNTX-Cbtx-c, cobrotoxin-c (UniProt: P59276, *N. kaouthia*); Hs_SαNTX-1, short neurotoxin 1 (UniProt ID: P68415, *H. schistosus*); and Lc_SαNTX-2, short neurotoxin 2 (UniProt ID: P10457, *L. colubrina*). The details analyses were provided in Supplementary File S1.

The general trend of M_2_R scores for respective α-NTXs is shown to be less than 0.3 (Figure 8) while all predicted epitopes feature multiplier HADDOCK score ratio (h) ≤1 (Maximum M_2_R = 1), suggesting α-NTXs are generally poor immunogens. The result is paralleled with the algorithm clustering of MHCII binders that showed the predomination of weak MHCII binders (Table 2). The accumulated overall M_2_R scores for each α-NTX were also estimated and shown descending from Oh_LαNTX-OH55(0.28) > Hc_LαNTX-1(0.27) > Nk_SαNTX-Cbtx-c(0.25) > Hs_SαNTX-1(0.22) > Nk_LαNTX-α-Cbtx(0.2) > Lc_SαNTX-2(0.11) (Figure 8).

**FIGURE 9:**
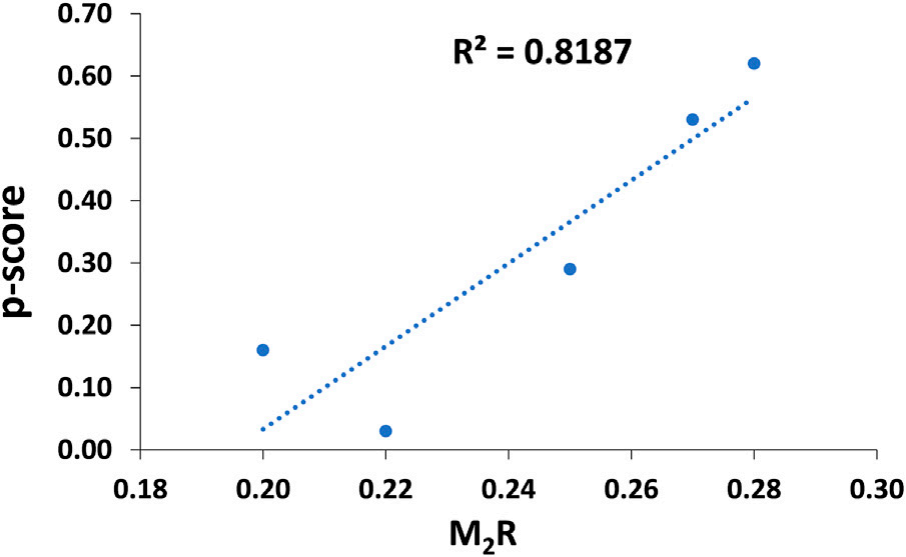
Coefficient of determination between empirical-based p-score and computed M_2_R scoring.

For comparative purposes, Table 3 illustrates the correlation study of reported commercial antivenoms on five selected α-NTXs examined in the present study. The p-score, defined as the relative potency of commercial antivenom for respective α-NTXs, is highly correlated (coefficient of determination, *R* ^2^ = 0.82) with M_2_R scoring in the present study (Figure 9). The p-score trend showed as: Oh_LαNTX-OH55(0.62) > Hc_LαNTX-1(0.53) > Nk_SαNTX-Cbtx-c(0.29) > Nk_LαNTX-α-Cbtx(0.16) > Hs_SαNTX-1(0.03). Except for the slight swapping order between Nk_LαNTX-α-Cbtx and Hs_SαNTX-1, the p-score generated is consistent with the M_2_R score in showing Hc_LαNTX-1 and Nk_SαNTX-Cbtx-c are more immunogenic compared to their homologous Hs_SαNTX-1 and Nk_LαNTX-α-Cbtx, respectively. The finding is however inconsistent with the earlier postulation where antigenicity and immunogenicity of LαNTXs are generally greater than SαNTXs, attributed to the slightly greater molecular size (Tan et al., 2016b; Wong et al., 2016; Tan et al., 2017). The present study showed a mixed observation that both LαNTXs and SαNTXs are generally low in antigenicity and immunogenicity, without clear distinction.

**TABLE 3:** Correlation of neurotoxin abundance and neutralization efficacy of its homologous antivenom.

Elapid Antivenom	*Ophiophagus hannah* monovalent antivenom (OHMAV)	*Naja kaouthia* monovalent antivenom (NkMAV)		Sea snake antivenom (SSAV)	
α-NTXs	Oh_LαNTX-OH55	Nk_LαNTX-α-Cbtx	Nk_SαNTX-Cbtx-c	^@^Hc_LαNTX-1	Hs_SαNTX-1
Relative abundance of α-NTXs in venom (%)	∼43.0^Ɨ^	30.9^#^	4.6^#^	11.9^@^	52.2^@^
Normalized potency (mg/g)	26.75^&^	4.89^#^	1.33^#^	6.35^#^	1.61^#^
p-score	0.62	0.16	0.29	0.53	0.03

The values were acquired from previous studies reported from the same laboratories: ^Ɨ^(Tan et al., 2015a); ^&^unpublished; ^#^(Tan et al., 2016b); ^@^(Tan et al., 2015b). p-score: Normalized potency of the respective antivenom/relative abundances of α-neurotoxin in the venom. ^@^LαNTX of *Hydrophis schistosus* was annotated to LαNTX of *Hydrophis curtus* (Hc_LαNTX-1) in the venom proteome study (Tan et al., 2015b).

On the other hand, the p-score shown in Table 3 also highlights the disproportionate link between the relative abundance of α-NTXs and the neutralization efficacy of antivenoms. The poor neutralization potency of antivenom against α-NTXs could be multifactorial (e.g., molecular size, relative abundance, amino acid composition, and peptide binding recognition by MHCII), in particular the amino acid composition as highlighted in the present study, contributing to the inadequate immunogenic capacity of α-NTXs in eliciting a strong humoral response during the immunization in an animal host. Furthermore, the antigenicity could have been impacted by its small size when competing for B-cell receptors in the context of a heterogenous immunogen mixture (Hunt et al., 1995).

To date, a number of innovative strategies for new antivenom production have been reported, such as the use of synthetic peptide antigen (de la Rosa et al., 2018), multiepitopes string (Ramos et al., 2016) and virus-displayed epitopes (Menzies et al., 2022) as immunogens. These strategies selected the toxins mainly based on targeted snake species and showed that it is possible to produce elapid antivenoms by replacing crude venom immunogens. In the future exploration of antivenom production, the immunoinformatic approach developed in this study can be applied to further strengthen the design of synthetic epitopes, thereby increasing the neutralization efficacy of antivenoms, especially in targeting the alpha-neurotoxins of elapid species.

## 4 Conclusion

In the present study, antigenicity tool and structure-based MHCII epitope predictor augmented with DM-editing determinant screening algorithm, collectively allude α-NTXs as poor antigen and immunogen. This is further supported by the relative immunogenicity of α-NTXs derived from the computed prediction that correlates strongly to the empirical data of *in vivo* neutralization efficacy of commercial antivenoms. Adding to that, most of the predicted binders feature non-optimal P1 anchor residues that greatly impede the formation of the MHCII-peptide complex with stronger affinity and stability. Overall, the outcome of this study suggests that both molecular size and amino acid composition are the main factors limiting the epitope configuration of α-NTXs as recognized by B-cell receptor (BCR) and T-cell receptor (TCR), resulting in a weaker humoral response in the animal host during immunization that applies a whole venom immunogen. The finding of the study provides insight into the low efficacy of elapid antivenoms against most elapid venoms which contain α-NTX as the principal toxins. Furthermore, DM-editing determinants could be incorporated as feature descriptors to the existing machine-learning-based predictors to enhance their accuracy in predicting MHCII epitopes while enabling the elucidation of immunodominant coverage of α-NTXs. This application has potential utility in the design and production of synthetic epitope immunogens for developing a better antivenom by applying various innovation strategies (Ramos et al., 2016; de la Rosa et al., 2018; Menzies et al., 2022).

## Data Availability

The original contributions presented in the study are included in the article/Supplementary Material, further inquiries can be directed to the corresponding author.
